# Continuous usage intention of mobile health services: model construction and validation

**DOI:** 10.1186/s12913-023-09393-9

**Published:** 2023-05-05

**Authors:** Li Nie, Brian Oldenburg, Yingting Cao, Wenjie Ren

**Affiliations:** 1 School of Management, Xinxiang Medical University, Xinxiang, Henan China; 2 Implementation Science Lab, Baker Heart and Diabetes Institute, Melbourne, VIC Australia; 3 School of Psychology and Public Health, La Trobe University, Melbourne, VIC Australia; 4 School of Allied Health, Human Service and Sport, La Trobe University, Melbourne, VIC Australia

**Keywords:** mHealth services, Continuous usage intention, E-health literacy, Subjective norm, Perceived usefulness

## Abstract

**Background:**

Mobile health (mHealth) services can not give full play to their value if only it is used in the short term, and their continuous usage can achieve better effects in health management. This study aims to explore the factors that affect continuous usage intentions of mHealth services and their mechanism of action.

**Methods:**

First, considering the uniqueness of health services and social environmental factors, this study constructed an extended Expectation Confirmation Model of Information System Continuance (ECM-ISC) to investigate factors that may influence the intention of continuous usage of mHealth services based on three dimensions, namely individual characteristics, technology and environment. Second, the survey method was used to validate the research model. The questionnaire items were derived from validated instruments and discussed by experts and data were collected both online and offline. The structural equation model was used for data analysis.

**Results:**

There were 334 avidity questionnaires through cross-sectional data and these participants had used mHealth services ever. The reliability and validity of the test model were good, in which Cronbach’s Alpha values of 9 variables exceeded 0.9, composite reliability 0.8, the average variance extracted value 0.5, and the factor loading 0.8. The modified model had a good fitting effect and strong explanatory power. It accounted for 89% of the variance in expectation confirmation, 74% of the variance in perceived usefulness, 92% of the variance in customer satisfaction, and 84% of the variance in continuous usage intention. Compared with the initial model hypotheses, perceived system quality was deleted according to the heterotrait-monotrait ratio, so paths related to it were deleted; perceived usefulness wasn’t positively associated with customer satisfaction, and its path was also deleted. Other paths were consistent with the initial hypothesis. The two new added paths were that subjective norm was positively associated with perceived service quality (*β* = 0.704, *P* < 0.001), and perceived information quality (*β* = 0.606, *P* < 0.001). Electronic health literacy (E-health literacy) was positively associated with perceived usefulness (*β* = 0.379, *P* < 0.001), perceived service quality (*β* = 0.200, *P* < 0.001), and perceived information quality (*β* = 0.320, *P* < 0.001). Continuous usage intention was influenced by perceived usefulness (*β* = 0.191, *P* < 0.001), customer satisfaction (*β* = 0.453, *P* < 0.001), and subjective norm (*β* = 0.372, *P* < 0.001).

**Conclusions:**

The study constructed a new theoretical model including E-health literacy, subjective norm and technology qualities to clarify continuous usage intention of mHealth services, and empirically validated the model. Attention should be paid to E-health literacy, subjective norm, perceived information quality, and perceived service quality to improve continuous usage intention of users and self–management by mHealth Apps managers and governments. This research provides solid evidence for the validity of the expanded model of ECM-ISC in the mHealth field, which can be a theoretical and practical basis for mHealth operators’ product research and development.

**Supplementary Information:**

The online version contains supplementary material available at 10.1186/s12913-023-09393-9.

## Background

MHealth services were defined as the use of mobile equipment such as mobile phones, patient testing equipment, personal digital assistants, and other wireless equipment that provided medical and public health operation services to improve healthcare processes, outcomes, and self-management of users [1]. In recent years, China's internet industry has made remarkable progress in digital infrastructure, economy, and governance, strongly promoting the development of mHealth. The standardized level of internet medical service continues to improve. The regulatory policy framework related to the internet medical field has been improved, guiding the standardized development of the Internet medical industry. By december 2022, the number of Internet medical users in China had reached 363 million, an increase of 64.66 million compared with that of december 2021, accounting for 34.0% of the total netizens [2]. And governments in several countries have endeavored to meet the urgent need to accelerate the adoption of mHealth apps. In Germany, an application called the Digital Health Act and certified health apps (DiGA) became a patient’s trusted digital assistant starting from summer 2021, and physicians can prescribe a certified act and many citizens living with chronic conditions already use free apps to improve their quality of life and track symptoms [3]. In the United Kingdom, the National Institute for Health and Care Excellence has published guidance about “Sleepio” on 20 May 2022, which is a digital self-help program and is recommended as a cost-saving option for treating insomnia and insomnia symptoms in primary care for people, and evidence showed Sleepio was more effective in reducing insomnia symptoms than the comparator [4]. The mHealth overcomes the traditional of offline medical treatment time of distance, time, and cost, especially when the COVID-19 pandemic prompts users to further change their medical consumption habits. The survey results of Yang et al. [5] showed that during the period, residents using the Internet for medical treatment accounted for 39.29%, and online appointments, registration, hospital doctor search, and payment accounted for more than 45%, meeting the needs of living far from medical institutions, online surgery appointment, less disease, avoiding contact with doctors, and communicating with doctors at any time. The capacities of prevention & control and diagnosis & treatment of medical institutions in remote areas has been improved. However, there are still some problems such as fewer core medical services of diagnosis, poor stickiness and serious homogeneity of services, and low users’ intention to pay, which lead to low continuous usage intention. A national survey showed [6] that 45.7% (427/934) of the users no longer used certain mHealth Apps after these Apps were downloaded. Vaghefi et al. [7] also found that most users only use it 4 times, and 25% use it only once after installation. Initial use was the only first step for the success of an information system, and protecting users’ continuous intention was more critical [8], because the effectiveness of health management based on a mHealth app was affected by user behavior, and short-term use could hardly achieve the expected goal of health management. Users’ low adoption and utilization rate has been a main hindrance to mHealth’s continuous development. So continuous usage of mHealth service is important for platforms, users and the whole medical system. When platforms improve operations and management, users can not only get more accurate and professional mHealth services but also reduce their time and cost of going to the hospital. It can cope with the contradiction between the shortage of medical resource supply and people’s increasing health needs [9]. Therefore, it is of great significance for both platforms and users, and it is essential to alleviate the imbalance of supply and demand of medical services and promote the construction of digital medical services for sustainable development.

With the acceleration of global digital health, mHealth services have been an effective way to address health. Sawyer et al. [10] analyzed smoking cessation apps could be acceptable and feasible, and tailored to the needs of people with schizophrenia through a non-systematic narrative review; Wang et al. [11] studied that mHealth interventions were a promising approach to improving diets of cancer survivors through meta-Analysis. These researches are carried out for a certain type of mobile service APP and adopt qualitative research methods are adopted. Meanwhile, since Bhattacherjee [12] first proposed the ECM-ISC model and applied it to online banking users, many scholars have verified the model in research of different mobile APP fields and combined it with other influencing factors to examine users’ continuous intention (Additional file 1). For example, Kim et al. [13] analyzed the antecedent factors affecting continuous intention to use online to offline accommodation app services, empirically verified them by applying the expectation-confirmation model, and showed that technicality, usefulness, and confirmation on perceived value and satisfaction were important in inducing continuous intention to use apps. Cheng [14] and Wang et al. [15] put forward an improved ECM-ISC model for nurses’ and college students’ continuous learning intention, and the latter added students’ initial intention and teacher guidance factor. The ECM-ISC model can be applied to gamification elements of a virtual community, the mechanism of enterprise brand value formation and characteristics and incentive types of the webcast on users' attitudes [16–18]. These studies of intention of continuous usage through the ECM-ISC model focus on accommodation, learning, and gaming communities, with little reference to mobile health services. Some studies on mHealth services Apps focused on the influencing factors of users' intention to adopt or patients' initial intention to use [19–21]. Also, some studies reviewed continuous usage for cost-effective effects to Glycemic Control of continuous usage of diabetes management Apps [22, 23], whereas only a few studies reviewed continuous usage of mHealth Apps and explained the mechanism. Zhang et al. [24] provided a multi-dimensional perspective from patient characteristics, medical care characteristics, and system quality to explore the factors of continuously using mobile medical Apps for medical consultation, without considering intermediate variables such as expectation confirmation and customer satisfaction. Song et al. [25] developed a model to measure the success of patients’ continuous use of mHealth services of chronic conditions, without exploring perspective health and subjective norm. Wang et al. [26] explored the impact path of users’ feelings induced by gamification on the intention of continued use of mHealth Apps through the ECM-ISC. Tian et al. [27] studied the ECM-ISC has found a solid theoretical basis for research into consumers’continuous usage of mHealth Apps, and added effort expectancy, social influence, and facilitating conditions of elders with chronic diseases.

A literature review reveals that the ECM-ISC model has laid a solid theoretical basis for research into consumers’ continuous use by adding some variables or combinations with other theoretical models. Nevertheless, few studies have applied this model in the field of mhealth services, and their studies are carried out from a certain level, which lacks a comprehensive analysis of factors from multiple perspectives of mhealth services. Therefore, this study adopted the system theory comprehensively considered the environment faced by mhealth services. Based on previous studies of technical factors, considering the uniqueness of mhealth service, we added the E-health literacy of individual characteristics and subjective norms of social environment. A comprehensive and integrated expanded ECM-ISC model including individual characteristics, technology, and social environment was constructed. That is, E-health literacy, platform service quality, information quality, and system quality were taken as antecedent variables. And social norm was incorporated into the model as a direct influence on the continuous usage intention of mhealth services. We explored how individual characteristics and platform quality affected the intention to continuous usage through intermediate variables such as perceived usefulness, expectation confirmation, and customer satisfaction, and whether social norms affected the intention. In addition, the paths with insignificant *P* values were deleted and two paths were added, based on the unreasonable fitting index of a part of the initial model, combined with the realistic characteristics of continuous use of mhealth services, and in strict compliance with the revision requirements of structural equation model. Thereby, this research expanded the ECM-ISC to make it more consistent with actual situations of mHealth services in an attempt to provide a better explanation of continuous intention, and empirically validated the factors that affected the continuous intention of mHealth Apps. It broads new scenarios for the application of models in the field of health services, and expands new boundaries for the application of health service theory. At the practical level, it explores the influencing factors for the continuous usage intention of mhealth services, the degree of impact of each factor, and their interrelationships, promoting the development of mhealth services in China, pushing forward managers and companies to improve service quality, and improving people's ability of self-health management. The specific three objectives are (1) preliminarily determining the factors that affect the continuous usage intention of mHealth services, (2) building a theoretical model to clarify the interaction between the influencing factors, and (3) empirically validating the mechanism of action among the various factors. The theoretical model is conducted by the following factors influencing continuous usage intention of mHealth Apps.

### Theory foundation and research hypotheses

This proposed model was drawn from the ECM-ISC model [12], which included four variables: expectation confirmation, perceived usefulness, customer satisfaction, and continuous usage intention of the information system (Additional file 2). In the ECM-ISC, the four variables were correlated with each other. Expectation confirmation and perceived usefulness were positively associated with customer satisfaction, expectation confirmation was positively associated with perceived usefulness, and customer satisfaction was positively associated with continuous usage intention. In the follow-up research, some scholars added specific variables to the basic model to make a fuller explanation. Some theoretical and empirical studies have proved that ECM-ISC model can predict and explain continuous usage intention in the mobile apps [28, 29]. They respectively extended some variables such as platform quality, users’ characteristics, and gamification etc. But they lacked overall consideration of different factors. Therefore, we constructed a new theoretical model to explore how individual characteristics, platform quality, and external environment affected continuous usage intention.

Hypotheses of perceived usefulness and expectation confirmation are as follows. Perceived usefulness refers to the degree of usefulness feeling. When users’ demand for mHealth service was high, their satisfaction increased, perceived usefulness was strong [30], and they would continue using it. Geffen [31] also verified that perceived usefulness affected continuous usage intention to adopt. Expectation confirmation refers to the different degrees of users’ feeling between post-use experiences and previous expectations. Studies have shown that the positive impact of expectation confirmation on perceived usefulness and satisfaction was very significant [12]. Customer satisfaction refers to the comparison of the initial expectation with their post-use experiences, that is, the degree to which user demands are met. Bhattacherjee [32] found that satisfaction had a positive association with continuous usage intention. Therefore, a summary of hypothesis statements is given below:

H1: Perceived usefulness is positively associated with satisfaction of mHealth services.

H2: Perceived usefulness is positively associated with the continuous intention of mHealth services.

H3: Expectation confirmation is positively associated with perceived usefulness of mHealth services.

H4: Expectation confirmation is positively associated with customer satisfaction of mHealth services.

H5: Customer satisfaction is positively associated with the continuous intention of mHealth services.

Hypotheses of individual characteristics of mHealth services are as follows. E-health literacy [33] refers to the users’ ability to obtain, evaluate, and use health information to meet health needs in the network environment and realize self-efficacy in health, information, and media. Zhang et al. [24] chose E-health literacy and health awareness as the variables when they studied individual characteristics, and found that health awareness was not related to the continuous usage intention and patients’ electronic health literacy affected the quality of the relationship and the continuous usage intention of mobile medical Apps. Patients with higher E-health literacy were more able to use websites for better interaction and obtained health information according to their demands, which increased self-efficacy and platform trust. Therefore, individual characteristics were measured by E-health literacy. Health Belief Model also proposed that user E-health literacy affected self-efficacy and self-management, which had a positive association with perceived services and perceived information [34, 35]. Therefore, a summary of hypothesis statements is given below:

H6a: E-health literacy is positively associated with the perceived service quality of mHealth.

H6b: E-health literacy is positively associated with the perceived information quality of mHealth.

H6c: E-health literacy is positively associated with the perceived system quality of mHealth.

H6d: E-health literacy is positively associated with the perceived usefulness of mHealth service.

Hypotheses of perceived qualities of mHealth services are as follows. The system success model includes three indicators: system quality, service quality, and information quality.System quality reflects the status and characteristics of mHealth services; service quality reflects responsiveness and reliability of mHealth services, in which responsiveness refers to timely response to users’ demands and reliability refers to the ability and accuracy to complete the promised service; and information quality refers to provide timely, accurate and complete information of mHealth services [36]. Shin [37] pointed out that system quality and information quality were the explanatory indicators of the success model of the system. Zhang [24] researched continuous usage intention in mHealth Apps and mobile government Apps [38], which established the system success model and verified that the three indicators positively and significantly affected the expectation confirmation. Therefore, a summary of hypothesis statements is given below:

H7a: Perceived system quality is positively associated with expectation confirmation of mHealth services.

H7b: Perceived service quality is positively associated with expectation confirmation of mHealth services.

H7c: Perceived information quality is positively associated with expectation confirmation of mHealth services.

Subjective norm refers to the social pressure that an individual performs or does not perform a certain behavior, which is related to the normative beliefs expected by others [25]. Users may change their beliefs and behaviors according to expectation and recommendation of their life circles, friends or important colleagues. Yan et al. [39] showed that self-fulfillment demands and social supports were positively related to users’ knowledge sharing behaviors when using mHealth services. Choi et al. [40] pointed out that subjective norm was an important basis for decision-making, and had a significant positive association with the adoption of online opinions. Subjective norm as the external environment variable significantly and positively affected the continuous usage intention of wearable devices. Therefore, the following hypothesis is posited:

H8: Subjective norm is positively associated with continuous usage intention of mHealth services.

**Fig. 1:**
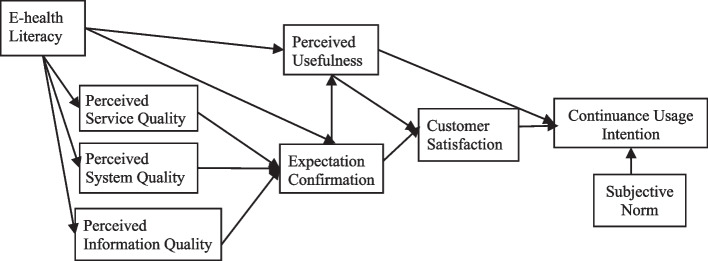
Influencing factors of continuous usage intention in the initial model of mHealth services

In the proposed theoretical model, combined with the actual situations of mHealth services, individual characteristics are expressed through E-health literacy, platform quality through perceived service quality, perceived system quality and perceived information quality, and social environment through subjective norm. There are 9 variables in the model represented. Five variables are independent: E-health literacy, perceived system quality, perceived service quality, perceived information quality, and subjective norm. The continuous usage intention is the dependent variable. The hypothetical relationships among the variables are shown in Fig. 1.

## Materials and methods

### Study setting and data collection

We added 5 variables in the ECM-ISC that affected mHealth services, so the model was extended by the variables from 4 to 9. These variables couldn’t be directly observed and were measured indirectly through 29 observation variables. At the same time, the influence relationship paths between the variables were set according to the research hypotheses proposed in the study. The questionnaire was completed through the professional questionnaire platform of Questionnaire Star, whose details were referred to Additional file 3. Convenience sampling was used in the survey. Before the formal survey, we conducted a pre-survey of 50 mHealth service users, modified, and adjusted the items based on the feedback results. To improve the variety of common methods, paper questionnaires and electronic questionnaires were adopted, which were distributed and received both through on-site and online surveys. We explored the factors of continuous usage intention of mHealth service. So adults who had used or were using mHealth service were eligible to participate in the study. We excluded participants who had never used the mHealth service through an item to filter out invalid samples. Respondents who chose ‘Yes’ would continue to answer, and those who chose ‘No’ would end the survey.

**Fig. 2:**
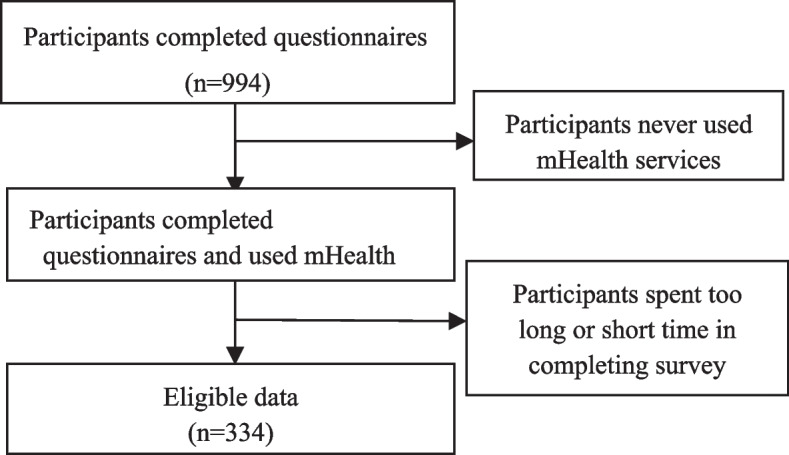
Flow diagram for the survey participants

A questionnaire consisting of two parts was developed in 2019. The first part was the basic demographic characteristics and the second one was the measurement items of the continuous usage intention of mHealth service. The item used a 5-point Likert scale from 1 (strongly disagree) and 5 (strongly agree). The questionnaire was composed of mature scales to ensure the reliability and validity of the measurement and then was discussed and validated by a team of 7 experts, including 3 health management experts, 2 health operation managers, and 2 medical information experts. The questionnaires were distributed in early november 2020 and lasted for one month through cross-sectional data. 994 copies of the valid questionnaire were collected which were filled in via online and offline channels. We chose the Baolong community in Hongqi District, Xinxiang City, Henan Province. As to online copies, we invited people to fill out the questionnaire through the community service center, and finally collected 674 valid copies. As to offline copies, we visited the community square of residential area in person to invite people to fill in the questionnaire. In order to avoid repetition we first asked if they had finished the questionnaire online, and then finally collected 320 valid copies. 566 of the 994 participants were excluded (Fig. 2) because they did not use mHealth services. According to the time spent on the pre-test questionnaire, in order to ensure the validity of the questionnaire, less time-consuming questionnaires were excluded. Depending on the screening questions, 334 valid questionnaires were finally obtained.

According to Barclay et al. [41], the minimum sample size was 10 times the number of questions or paths in the measurement model to ensure the robustness of the sample estimates. The number of questions and paths in the study was 29 and 10, so the minimum number of sample was 200. 334 valid samples were collected, which met the requirements of sample analysis.

### Data analysis and measurement equations

To study the influence of the individual, technology, and environment on the continuous usage intention of mHealth services, we constructed a structural equation model of the relationship. The model could systematically test hypotheses, interpret the relationship among variables based on their covariance matrix as a whole and address collinearity caused by the high correlation in variables, which was an important tool for analyzing complex causal relationships among multiple latent variables [42]. Moreover, it could reflect the direct and indirect relationship of the variables. It had been widely used in the analysis of health management [15, 25]. The structural equation model was divided into two parts: measurement equation and structural equation. The data was analyzed below using IBM SPSS v26.0 and IBM AMOS v26.0 (IBM, Armonk, NY, USA).

The measurement equation was tested by reliability and validity. The reliability was composed of the internal consistency coefficient (Cronbach's Alpha) and the composite reliability. It was generally considered that the coefficient of the two variables was quite good at 0.7–0.8. More than 0.8 meant it was very good [43]. For reflective constructs, reliability was also assessed by the collinearity statistics, that is, outer variance inflation factor values. If it was less than 10, there was no multicollinearity between variables. The validity was composed of content validity and construct validity. The questionnaires in this study were all derived from mature scales to ensure the consistency between variables and measurement items, which had good content validity after expert review and pre-investigation. Therefore, we mainly tested the construct validity of the questionnaire, which consisted of convergent and discrimination validity. Convergent validity was measured by average variance extracted, factor loading, and heterotrait-monotrait ratio. It was generally believed that the average variance extracted value was greater than 0.5, the factor loading was greater than 0.7, and the scale had a higher degree of aggregation. Discrimination validity was measured by the correlation among latent variables. When the average variance extracted square root value of each factor was greater than the maximum value of the correlation coefficient between this factor and other factors, the discrimination validity was relatively high [44]. Thresholds of the heterotrait-monotrait ratio are 0.85, and 0.9 when two concepts are close [45].

As for the impact of demographic variables on the results, single factor analysis of variance was conducted with continuous usage of mHealth service as the dependent variable and for demographic variables, namely gender, age, education, and health levels, as the independent variables, to determine the control variables entering the structural equation model.

## Results

### Characteristics of the participants

Table 1 provides the demographic characteristics of 334 participants. There were more female participants (194/334, 58.1%) than male participants (140/334, 41.9%). Approximately 85.4% (285/334) of participants were in the age group of 18–40 years, about 83.5% (279/334) had an education level of bachelor’s degree or above, 53% (177/334) were good in self-recognition of health status and 66.8% (223/334) had a usage frequency of less than 1 time a week.

**Table 1:** Characteristics of the participants (*N* = 334)

Characteristics	Value, n (%)
Sex	
Male	140 (41.9)
Female	194 (58.1)
Age, years	
18–30	226 (67.7)
31–40	59 (17.7)
41–50	40 (12)
> 50	9 (2.7)
Education	
Junior college and below	55 (16.4)
Bachelor’s degree	206 (61.7)
Postgraduate degree	59 (17.7)
Doctoral degree	14 (4.2)
Self-recognition of health status	
Poor	19 (5.7)
Fair	138 (41.3)
Good	177 (53)
Usage frequency	
More than 4 times a week	27 (8.1)
1–3 times a week	84 (25.1)
less than 1 time a week	223 (66.8)

### Test of reliability and validation of model

For the reliability test, Cronbach's Alpha values of 9 latent variables all exceeded 0.9, which would all decrease after deleting a measurement item, indicating that the questionnaire had good consistency reliability. The composite reliability of these variables exceeded 0.8 two of which were above 0.9, indicating that the questionnaire had good combined reliability (Table 2). For the validity test, the average variance extracted value of each variable was above 0.5, and the factor loading was above 0.8. Some values were above 0.9 according to the initial heterotrait-monotrait ratio, so perceived system quality, the third item of expectation confirmation and the third item of customer satisfaction were deleted. There were only five values more than 0.85 which were close in the initial model and the others were less than 0.85 (Additional file 4). They confirmed convergent validity (Table 2); the average variance extracted square root of each variable was greater than its squared correlation with other variables (Table 3), which confirmed discrimination validity. Therefore, the model had good convergent validity and discrimination validity, and the maximum value of variance inflation factor was 6.428, so there was no multicollinearity in the model.

**Table 2:** Test reliability and convergent validity of variables

Variables	Code	Cronbach's a value	Value after deleting item	Normalized factor loading	Composite Reliability	Average variance extracted
E-health Literacy (EH)	EH1	.928	.909	0.882	0.929	0.765
	EH2		.891	0.932		
	EH3		.914	0.836		
	EH4		.910	0.846		
Perceived Usefulness (PU)	PU1	.940	.921	0.896	0.940	0.796
	PU2		.921	0.884		
	PU3		.917	0.907		
	PU4		.925	0.881		
Perceived Information Quality (IQ)	IQ1	.914	.865	0.903	0.812	0.590
	IQ2		.856	0.91		
	IQ3		.909	0.847		
Perceived System Quality (SQ)	SQ1	.913	.854	0.904	0.806	0.581
	SQ2		.888	0.829		
	SQ3		.881	0.905		
Perceived Service Quality (SEQ)	SEQ1	.928	.889	0.888	0.823	0.608
	SEQ2		.899	0.913		
	SEQ3		.899	0.901		
Expectation Confirmation (EC)	EC1	.915	.872	0.867	0.807	0.583
	EC2		.862	0.865		
	EC3		.902	0.911		
Customer Satisfaction (CS)	CS1	.961	.938	0.953	0.859	0.670
	CS2		.954	0.926		
	CS3		.937	0.955		
Subjective Norm (SN)	SN1	.934	.923	0.864	0.829	0.618
	SN2		.895	0.917		
	SN3		.893	0.941		
Continuous Usage Intention (CU)	CU1	.934	.925	0.881	0.831	0.621
	CU2		.878	0.938		
	CU3		.908	0.91

**Table 3:** Correlation coefficient between square root of average variance extracted and latent variable

Constructs	E-health Literacy	Perceived Usefulness	Perceived Information Quality	Perceived System Quality	Perceived Service Quality	Expectation Confirmation	Customer Satisfaction	Subjective Norm	Continuous Usage Intention
E-health Literacy	0.875								
Perceived Usefulness	0.461	0.892							
Perceived Information Quality	0.373	0.528	0.768						
Perceived System Quality	0.337	0.519	0.607	0.762					
Perceived Service Quality	0.333	0.498	0.599	0.625	0.780				
Expectation Confirmation	0.358	0.499	0.562	0.565	0.58	0.763			
Customer Satisfaction	0.356	0.505	0.585	0.577	0.607	0.662	0.818		
Subjective Norm	0.314	0.473	0.524	0.527	0.581	0.598	0.616	0.786	
Continuous Usage Intention	0.333	0.508	0.552	0.555	0.558	0.588	0.601	0.627	0.788

### Initial and modified model of structural equation

The initial model was tested according to the requirements of the critical value of the adaptation standard. The fitting indexes of the model were not ideal and the values of χ2/DF, RMSEA and RMR were higher than the standard value (Table 4). When the fitting indexes of the initial model were unsatisfactory, the model could be modified by deleting or adding paths. For example, Liu and Zhang [46] analyzed the decision-making behavior of consumers on takeaway mobile Apps by modified structural equation model, and Hong et al. [47] studied public governance, incentive structure, and sustainable development of special economic zones by a case study based on modified structural equation model. So this modified model was structured by deleting paths of the insignificant *P* value and re-estimating the possibly related paths [44], and the initial model was corrected by modification index, which expanded the model in line with the practical usage of mHealth services, following the principle of starting from the largest index and only adding a path each time. At the same time, the results of single factor analysis indicated that there only existed a significant difference in continuous usage of mHealth service among the subjects at different health levels, so health level as a control variable was included in the modified model.

**Table 4:** The initial model and the modified model fitting index value

Model Fitting Indices	χ2/DF	RMSEA	NFI	IFI	TLI	CFI	GFI	AGFI	RMR	PGFI	PNFI
Adaptation standard	1–3	< 0.08	> 0.9	> 0.9	> 0.9	> 0.9	> 0.8	> 0.8	< 0.05	> 0.5	> 0.5
Initial value	3.207	0.081	0.918	0.942	0.933	0.942	0.857	0.821	0.142	0.686	0.798
Model value	2.327	0.063	0.935	0.926	0.956	0.962	0.875	0.845	0.05	0.705	0.817

χ 2/d.f. chi-squared divided by degrees of freedom; RMSEA, root mean square error of approximation; *NFI* normalized fitting index, *IFI* modified fitting index, *TLI* non-normal fitting index, *CFI* comparative fit index, *GFI* goodness-of-fit index, *AGFI* adjusted goodness-of-fit index

### Common method bias test

Common method bias was verified by the common factor that was added as a latent variable to the revised result equation model [48]. The new model was compared with the model without the added common method factor and analyzed whether it had a significant change in the fitting index. If RMSEA and SRMR decreased by more than 0.05, and CFI and IFI increased by more than 0.1, it could prove that there was no serious common method bias. In the model with the common method factor, the RMSEA value, SRMR value, CFI value and TLT value was 0.058, 0.020, 0.973, and 0.966 respectively. In the other model, the RMSEA value, SRMR value, CFI value and TLT value was 0.064, 0.023, 0.964, and 0.959 respectively, so changes in four indexes were insignificant which could be confirmed that the common method bias in the sample data was not significant, and the results of the modified model were credible.

### Modified structural model and hypotheses testing

According to the result of the heterotrait-monotrait ratio, perceived system quality were deleted, so the paths H6c and H7a related to it were not included in the initial model. Some fitting indexes in the initial model did not meet the standards, such as χ2/DF, RMSEA, and RMR(Table 4). Moreover, *P* value of the path H1 was 0.058, which was not significant. Specifically, perceived usefulness was not positively associated with customer satisfaction, so the path H1 was deleted in the modified model. After many experiments, we got the modified model in which two paths were added. Compared with the fitting standard, all fitting indexes of the modified model met the critical value requirements and research demands and had good explanatory power (Table 4).

**Fig. 3:**
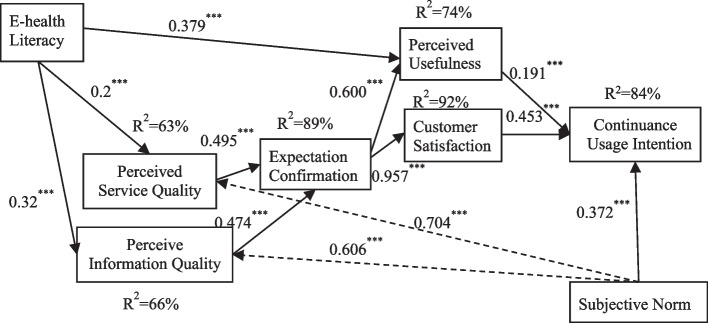
Structural equation model and path Note: *** means significant at the 0.001 level, R2 is squared multiple correlations

Compared with the initial hypotheses (Fig. 1), three paths, namely H1, H6c, and H7a,were deleted, which were the paths. Two paths were added according to the modification index (Fig. 3, Table 5), in which subjective norm was positively associated with perceived service quality and perceived information quality. According to the path correlation coefficient (Fig. 3), the stronger path was the effect of subjective norms on perceived service quality (0.704) and perceived information quality (0.606), perceived information quality on expectation confirmation (0.474), and expectation confirmation on perceived usefulness (0.600) and users’ satisfaction (0.957), in which the correlation coefficients were above 0.5. For the explanatory degree of the revised model, latent variables of expectation confirmation, perceived usefulness, customer satisfaction, and continuous usage intention were 89%, 74%, 92%, and 84% respectively, which confirmed the accuracy of the model (Fig. 3).

**Table 5:** Modified model path coefficient and hypotheses test results

Hypotheses	Variable relationship	Standardized path coefficient	95% confidence intervals	Standard error	Critical ratio	*P* value
			Lower Upper			
H2	PU → CU	0.191	0.197 0.528	0.049	4.149	***
H3	EC → PU	0.600	0.432 0.746	0.047	12.591	***
H4	EC → CS	0.957	0.926 0.984	0.035	29.213	***
H5	CS → CU	0.453	0.270 0.654	0.054	8.283	***
H6a	EH → SEQ	0.200	0.086 0.322	0.044	4.585	***
H6b	EH → IQ	0.320	0.202 0.445	0.045	7.005	***
H6d	EH → PU	0.379	0.229 0.539	0.040	8.959	***
H7b	SEQ → EC	0.495	0.077 1.745	0.074	6.250	***
H7c	IQ → EC	0.474	-0.991 0.842	0.076	6.017	***
H8	SN → CU	0.372	0.197 0.528	0.038	8.736	***
Added path1	SN → IQ	0.606	0.491 0.719	0.042	13.035	***
Added path2	SN → SEQ	0.704	0.590 0.798	0.400	16.079	***

H1: Perceived usefulness is positively associated with satisfaction of mHealth services

H6c: E-health literacy is positively associated with perceived system quality

H7a: Perceived system quality is positively associated with expectation confirmation

H2: Perceived usefulness is positively associated with continuous intention

H3: Expectation confirmation is positively associated with perceived usefulness

H4: Expectation confirmation is positively associated with customer satisfaction

H5: Customer satisfaction is positively associated with continuous intention

H6a: E-health literacy is positively associated with perceived service quality

H6b: E-health literacy is positively associated with perceived information quality

H6d: E-health literacy is positively associated with perceived usefulness

H7b: Perceived service quality is positively associated with expectation confirmation

H7c: Perceived information quality is positively associated with expectation confirmation

H8: Subjective norm is positively associated with continuous usage intention of mHealth services

Added path1: Subjective norm is positively associated with perceived service quality

Added path2: Subjective norm is positively associated with perceived service quality

^***^ means significant at the 0.001 level

## Discussion

### Principal findings and interpretations

This study constructed an expanded ECM-ISC model of continuous usage intention of mHealth service from three dimensions. The measurement model had good reliability and validity. After the structural equation model was revised, the ideal fitting effect was achieved. 10 paths passed the test in 13 initial hypothesis paths (Table 5). At the same time, two new paths were discovered.

Unlike the initial model, perceived system quality was deleted, because it is too relevant to perceived information quality and perceived service quality. In addition, we found that perceived service quality and perceived information quality positively affected expectation confirmation. But many mHealth Apps are currently provided by information or health technology companies, only a small part of which could set personalized reminders and provide professional information services [49], which didn’t meet the demands of users for deep-level health self-management.

Another attention should be paid to the important role of E-health literacy in the continuous usage intention of mHealth services, which has a positive impact on the perceived information quality, perceived service quality, and perceived usefulness which indirectly affects continuous usage intention. Song et al. [25] proposed that E-health literacy should be added as a latent variable in the model of continuous usage of patient health services. Users with higher E-health literacy could better interact with the mobile medical Apps to obtain the health information they neeed, increasing their trust in and satisfaction with the apps [24]. Tan [50] pointed out the main goal was to improve users' E-health literacy in his research on health information services provided by public libraries in the United States. Gao and Zhang [51] showed that E-health literacy affected the online health information behavior of the elderly.

We found that E-health literacy not only indirectly affected the continuous usage intention of mHealth services, but also directly affected perceived information quality and perceived service quality, so it was necessary to improve users’ E-health literacy. Two new paths associated with subjective norm were added. It indicated that subjective norm not only directly affects usage continuous intention, but also indirectly affects it through intermediate variables. This is consistent with planned behavior theory [52], which put forward subjective norm that affected intention and behavior. Zhao et al. [28] showed that subjective norm positively affected the continuous usage intention of wearable devices.

### Theoretical implications and practical implications

This study will present the complete process of “finding problems, proposing hypotheses, empirical research, and model revision”. In particular, through the revision, we have carried out an in-depth exploration of the relationship between latent variables. Most of the previous studies [24, 25] only performed fitness tests and path analyses on the null hypotheses of the model deleting insignificant paths, and did not make model corrections. Through experiments, this study reasonably deleted and increased the hypothetical paths of the original model. On one hand, it improves the fit and explanatory power of the model, On the other hand, the revised model suggests that we can discover hypotheses that were not initially noticed and find new relationships between variables, which can provide new key evidence and methodological reference for other scholars' research.

Different from the variable setting of the previous model, the three dimensions of individual characteristics, technology and social environment were comprehensively expanded in the ECM-ISC model. Song et al. [25] introduced individual health in continuous usage intention of mHealth services of patients, and Zhang considered individual health literacy for medical consultations, but they both did not consider the impact of subjective norm. Liang et al. [53] set subjective norm, but did not set individual characteristics when studying users' reading intention of wearable devices. From the theoretical perspective, compared with a single perspective, the integrated model can provide more explanations for the intention of continuous usage and expand its application in health service management and information. From the practical perspective, it provides inspiration for managers and mHealth service enterprises to formulate digital health incentives and implement policies from multiple perspectives. Platform enterprises should fully understand and tap the service needs of users, jointly developed and operated by health experts and clinicians, which can improve the professionalism of health services, provide more accurate health monitoring to avoid homogenous competition [33, 54]. Furthermore, they can regularly launch different subjects and activities through frequently used apps and multi-channel social platforms to attract users' attention and improve sustainable utilization.

Different from the previous empirical results, we found that E-health literacy and subjective norms indirectly affect continuous usage intention by affecting perceived service quality and perceived information quality. In the future, firstly, operators can encourage users to actively participate in online activities by offering free users’ health education knowledge discussions, Apps use guides, and consulting services. At the same time, in the community comprehensive service center, the manager promotes the mHealth science distribution project and the application of intelligent technology ability training and other publicity and education activities, to timely disseminate health management knowledge and improve people's E-health literacy. Secondly, we should focus on creating a diversified mHealth service support pattern. Subjective norms are the key factors affecting the continuous usage of mHealth services and people are likely to be recommended, evaluated, and commented by relatives, friends, colleagues and other people around them to change their attitudes and behaviors. Therefore, mHealth services can expand education, publicity, and promotion through community education institutions, community parties, service centers, grass-roots medical and health service institutions, government websites, radio and television programs, and other channels, to provide all-around support.

### Limitations and future research

This study has made new explorations on the continuous usage intention of mHealth services, but there are some shortcomings. Firstly, the results were significant to the path of H7c (Table 5), that is, perceived information quality was positively associated with expectation confirmation, but the upper and lower 95% confidence intervals contained the zero, perhaps due to the small sample size, we will try to expand the sample size in future research. Besides, since users of certain mobile service Apps were limited by the number of samples, the collection was not carried out according to the types of mHealth services. With the development of digital healthcare, certain types of App research can be selected to improve the pertinence of the results. Secondly, the process of questionnaire development can be more scientific, the sampling method could be improved and the object of the study can be targeted at specific groups of people. In future research, the process of questionnaire development can adopt the Delphi method, which can eliminate the influence of expert authority, and the results are more objective and credible. Besides, considered factors such as convenience, feasibility of implementation, cost, and availability of data, convenience sampling is used in the study, and related methods of probability sampling can be used in subsequent studies to improve the scientific nature of the study. In addition, the elderly, especially patients with chronic diseases, are in more need of health information. Studying their continuous usage intention is more positive for the promotion of online and offline health management services. Thirdly, given the uniqueness of mHealth services, more factors can be added in the later study, such as health status and privacy. And the mediating effects such as trust can also be discussed to further improve the scientificity and feasibility of the model [55–58].

## Conclusions

The research comprehensively considers three dimensions, namely individual, technology and environment, and builds an expanded ECM-ISC-ISC model of continuous usage intention of mHealth services. Through questionnaire surveys and empirical model corrections, perceived service quality, perceived information quality, subjective norm, and E-health literacy are important factors that affect continuous usage intention of mHealth services, and perceived system quality no longer affects continuous usage intention of mHealth services. The research has enriched and expanded the ECM-ISC model in terms of the setting of latent variables and the scope of application. In the future, a combination of questionnaire surveys and model can be used to conduct studies on certain types of health Apps or the elderly and patients with chronic diseases.

The results of the empirical analysis have positive implications for mHealth service providers and the government. Firstly, health service providers could provide more professional and personalized services to avoid homogeneous competitions by adding health experts and clinicians to their team. Secondly, attention should be paid to the feedback of users to improve their satisfaction and motivate them to spontaneously promote health information. Finally, it is necessary to improve the E-health literacy of users through online and offline service tutorials, consultation, and other methods to increase the stickiness of Apps use. After COVID-19, many people attach great importance to mHealth services as a part of public services. In additon, the government should continue to introduce digital medical policies and regulations to create a good social digital atmosphere, and improve subjective norm.

## Supplementary Information

**Additional file 1: Table 1.** Studies based on model of ECM-ISC.

**Additional file 2: Figure 1.** ECM-ISC Model.

**Additional file 3: Table 2.** Research scale and measurement items of factors affecting Continuance Usage Intention.

**Additional file 4: Table 3.** HTMT Analysis results.

## Data Availability

The datasets generated and analyzed during the current study are not publicly available because of the National Social Science Fund which states that data is made available to the public domain after 24 months. In the meantime, the data can be availed from the project leader, Li Nie: email 653,371,623@qq.com, upon reasonable request.

## Abbreviations

MHealth: Mobile health
E-health literacy: Electronic health literacy
ECM-ISC: Expectation Confirmation Model of Information System Continuance
