# Supplementary material for: Continuous usage intention of mobile health services: model construction and validation
Source: BMC Health Serv Res. 2023 May 5;23:442. doi: 10.1186/s12913-023-09393-9 (PMC10159674; doi:10.1186/s12913-023-09393-9)
Supplement: Supplementary file 1 — Additional file 1: Table 1. Studies based on model of ECM-ISC. [file 12913_2023_9393_MOESM1_ESM.docx]

Additional file 1

Table1 Studies based on model of ECM-ISC

| Object | Findings | Literature resources |
| --- | --- | --- |
| Online banking users | Cognitive beliefs and affect influences one's intention to continue using information systems, and users' continuance intention is determined by their satisfaction and perceived usefulness. User satisfaction, in turn, is influenced by their confirmation of expectation and perceived usefulness. | Bhattacherjee A. [12] |
| Nurses’ continued blended e-learning intention | The expectation confirmation has a significant influence on users’ perceived usefulness and satisfaction and that satisfaction had the greatest influence on users’ continuance use intention. | Cheng, Y.-M[14] |
| College students’ continued e-learning intention | Two latent variables were added, which were students’ initial intention and teacher guidance factor toward E-Learning Space | Wang W et al [15] |
| Gamification elements on Users' Willingness to Continue Using | Two latent variables were added, which were society and immersion in Interest-based Virtual Communities. | Feng Y, L et al [16] |
| Mechanism of enterprise brand value formation | Based on the fuzzy sentiment of online reviews，it verified the brand conversion intention and brand conversion type of cell phones by example calculation through loyalty theory and perceived value theory. | Ma Y. [17] |
| Influence of characteristics and incentive types of webcast on users' attitudes | The explicit stimulation of webcast has significant positive effects on users’ cognitive attitude, emotional attitude, and behavior attitude; the interaction of live network characteristics and incentive types has a significant positive impact on users’ cognitive attitude and emotional attitude. | Lin S et al [18] |
| Mobile Health Services of Chronic Conditions | The model contains 7 theoretical constructs: information quality, service quality, perceived usefulness, user satisfaction, perceived health status, and continuous use intention. | Song T et al [25] |
| The Impact of Gamification Induced Users' Feelings | the path and mechanism of users' feelings regarding autonomy, competence, and relatedness generated during interactions with different gamification elements promote the continued use of mHealth apps. | Wang T et al [26] |
| MHealth Continuance Intention of Elders with Chronic Diseases | Effort expectancy, performance expectancy, social influence and facilitating conditions can directly and significantly influence continuance intention. Among them, performance expectancy can directly influence continuance intention in the most significant way through ECM-ISC and UTAUT model. | Tian, et al [27] |
