# Supplementary figures and images for: Continuous usage intention of mobile health services: model construction and validation
Source: BMC Health Serv Res. 2023 May 5;23:442. doi: 10.1186/s12913-023-09393-9 (PMC10159674; doi:10.1186/s12913-023-09393-9)

Additional file 2


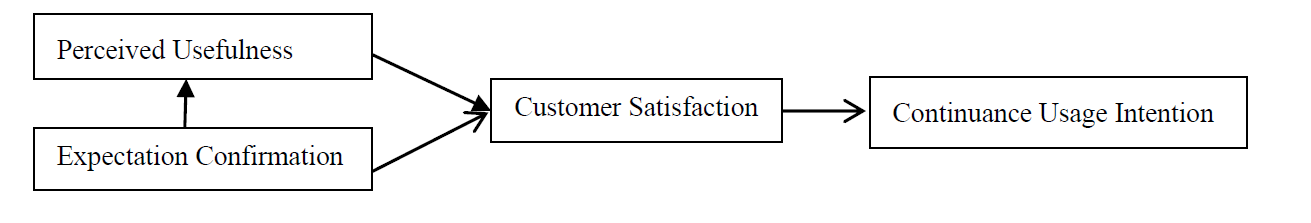


Figure 1. ECM-ISC Model.

Supplement: Supplementary file 2 — Additional file 2: Figure 1. ECM-ISC Model. [file 12913_2023_9393_MOESM2_ESM.docx]
