# Supplementary material for: Continuous usage intention of mobile health services: model construction and validation
Source: BMC Health Serv Res. 2023 May 5;23:442. doi: 10.1186/s12913-023-09393-9 (PMC10159674; doi:10.1186/s12913-023-09393-9)
Supplement: Supplementary file 3 — Additional file 3: Table 2. Research scale and measurement items of factors affecting Continuance Usage Intention. [file 12913_2023_9393_MOESM3_ESM.docx]

**Additional file 3**

Table 2. Research scale and measurement items of factors affecting Continuance Usage Intention.

| Variable | Code | Item | Literature resources |
| --- | --- | --- | --- |
| E-health Literacy  （EH） | EH1 | I know where I can get useful health resources information on the web | Norman C D, Skinner H A[34] |
|  | EH2 | I know how to use the health resources information I get from online to help myself |  |
|  | EH3 | I can distinguish between a high and low quality health resource information on the Internet |  |
|  | EH4 | I feel confident about using online information to make health-related decisions |  |
| Perceived Usefulness  （PU） | PU1 | I can get the knowledge or information I want by using mHealth services | Gefen D et al[31]， |
|  | PU2 | I have improved my access to knowledge or information by using mHealth services |  |
|  | PU3 | I can better manage my health by using mHealth services |  |
|  | PU4 | I think mHealth services are very useful |  |
| Information Quality（IQ） | IQ1 | MHealth services update information timely and provide users with the latest information | Bhattacherjee A [32]  Zhang Hai, Yao Ruihong [38] |
|  | IQ2 | MHealth services provide comprehensive and professional information required for users |  |
|  | IQ3 | MHealth services provide accurate information |  |
| System Quality  （SQ） | SQ1 | MHealth services have reliable and stable performance |  |
|  | SQ2 | MHealth services is easy to access |  |
|  | SQ3 | MHealth services can flexibly adapt to the various information needs of users |  |
| Service Quality  （SEQ） | SEQ1 | I will give an excellent evaluation of mHealth services |  |
|  | SEQ2 | MHealth services are of high quality |  |
|  | SEQ3 | MHealth services are excellent |  |
| Expectation  Confirmation  （EC） | EC1 | The benefits of using mHealth services are greater than I expected | Hu J M, Zhang Y[33] |
|  | EC2 | MHealth services provide more services than I expected |  |
|  | EC3 | Totally my overall expectations for mobile health services have been met |  |
| Customer  Satisfaction  （US） | CS1 | I am very satisfied with mHealth services | Bhattacher jee A[32] |
|  | CS2 | I am very satisfied with mHealth services experience |  |
|  | CS3 | In general, I am very satisfied with mHealth services |  |
| Subjective Norm（SN） | SN1 | Quite a few people I know are using mHealth services | Zhang Min et al [24] |
|  | SN2 | Quite a few people I know think using mHealth services is a good idea |  |
|  | SN3 | Quite a few people I know think I should continue using mHealth services |  |
| Continuance Usage Intention  （CU） | CU1 | I am willing to continue using mHealth services in the future | Zhang Hai, Yao Ruihong [38] |
|  | CU2 | I will often use mHealth services in the future |  |
|  | CU3 | I am willing to recommend mHealth services to my friends around |  |
