# Supplementary material for: Continuous usage intention of mobile health services: model construction and validation
Source: BMC Health Serv Res. 2023 May 5;23:442. doi: 10.1186/s12913-023-09393-9 (PMC10159674; doi:10.1186/s12913-023-09393-9)
Supplement: Supplementary file 4 — Additional file 4: Table 3. HTMT Analysis results. [file 12913_2023_9393_MOESM4_ESM.docx]

Additionbal file 4

Table 3. HTMT Analysis results.

|  | EH | PU | IQ | CS | CU | EC | SN | SEQ |
| --- | --- | --- | --- | --- | --- | --- | --- | --- |
| EH |  |  |  |  |  |  |  |  |
| PU | 0.695 |  |  |  |  |  |  |  |
| IQ | 0.572 | 0.806 |  |  |  |  |  |  |
| CS | 0.517 | 0.721 | 0.857 |  |  |  |  |  |
| CU | 0.493 | 0.760 | 0.830 | 0.881 |  |  |  |  |
| EC | 0.525 | 0.747 | 0.832 | 0.895 | 0.849 |  |  |  |
| SN | 0.437 | 0.635 | 0.712 | 0.814 | 0.834 | 0.751 |  |  |
| SEQ | 0.497 | 0.727 | 0.889 | 0.860 | 0.819 | 0.824 | 0.769 |  |
